# Role of IL-17 family cytokines in the progression of IPF from inflammation to fibrosis

**DOI:** 10.1186/s40779-022-00382-3

**Published:** 2022-05-12

**Authors:** Yun-Juan Nie, Shuo-Hua Wu, Ying-Hua Xuan, Gen Yan

**Affiliations:** 1 Department of Basic Medicine, Wuxi School of Medicine, Jiangnan University, Wuxi, 214000 Jiangsu China; 2 Department of Radiology, The Second Affiliated Hospital, Medical College of Shantou University, Shantou, 515000 Shandong China; 3 Department of Basic Medicine, Xiamen Medical College, Xiamen, 361000 Fujian China; 4 Department of Radiology, The Second Affiliated Hospital of Xiamen Medical College, Xiamen, 361000 Fujian China

**Keywords:** Interleukin-17 (IL-17) family, IL-17 receptor, Inflammation, Idiopathic pulmonary fibrosis

## Abstract

Idiopathic pulmonary fibrosis (IPF) is a fatal chronic interstitial lung disease with no established treatment and is characterized by progressive scarring of the lung tissue and an irreversible decline in lung function. Chronic inflammation has been demonstrated to be the pathological basis of fibrosis. Emerging studies have revealed that most interleukin-17 (IL-17) isoforms are essential for the mediation of acute and chronic inflammation via innate and adaptive immunity. Overexpression or aberrant expression of IL-17 cytokines contributes to various pathological outcomes, including the initiation and exacerbation of IPF. Here, we aim to provide an overview of IL-17 family members in the pathogenesis of IPF.

## Background

Idiopathic pulmonary fibrosis (IPF) is a progressive and ultimately fatal disease characterized by irreversible scarring and progressive decline of lung function, with a median survival time of 2–3 years following diagnosis [1]. The pathogenesis underlying IPF is poorly understood. Increasing evidence suggests that it results from abnormal wound healing following repetitive alveolar injury, accompanied by chronic inflammation associated with various types of inflammatory cells [2, 3]. Additional critical mechanisms associated with IPF progression are damage and apoptosis of alveolar epithelial cells and proliferation and differentiation of fibroblasts to secrete the extracellular matrix [4].

The interleukin (IL)-17 family is composed of six members, IL-17A, IL-17B, IL-17C, IL-17D, IL-17E, and IL-17F, according to homology-based cloning analysis. To date, most IL-17 family members have been shown to be produced by numerous cell types; they participate in a wide range of inflammatory diseases, including asthma, pneumonitis, and pulmonary fibrosis [5]. These cytokines act on endothelial and epithelial mesenchymal lineages and immunocytes to induce the secretion of cytokines and chemokines, such as granulocyte macrophage-colony stimulating factor (GM-CSF), tumor necrosis factor-α (TNF-α), IL-1β, and IL-18 [6]; exert a key function in the migration and differentiation of neutrophils in the lungs [7]; and contribute to airway remodeling by promoting the production of the profibrotic mediators IL-6 and IL-11 by fibroblasts [8–12]. The prototypical IL-17 family member IL-17A plays a key role in various inflammatory conditions by promoting the expression of inflammatory factors, including cytokines, chemokines, matrix metalloproteinases, and acute phase proteins. Overexpression or abnormal activity of IL-17A under pathological conditions can drive pulmonary fibrosis [13]. IL-17B is a less-characterized member of the IL-17 family. A recent study demonstrated that IL-17B induced the production of proinflammatory cytokines by inducing downstream signaling molecules through IL-17 receptor A (IL-17RA) and IL-17 receptor B (IL-17RB), which promote Th17 cell differentiation or neutrophil recruitment and activation to drive bleomycin (BLM)-induced pulmonary fibrosis progression. IL-17E has been demonstrated to play an analogous and crucial role in accelerating BLM-induced pulmonary fibrosis. IL-17C and D have been reported to play key roles in inflammatory pathologies, including lung inflammation; however, there is little direct evidence for their role in IPF [14, 15]. In this review, we summarize the current knowledge regarding the biological functions of IL-17 family cytokines and their involvement in the pathogenesis and progression of IPF.

## Biological characteristics of IL-17 family cytokines

IL-17 was first identified in 1993 [16, 17], and in the following years, five other IL-17 family members, IL-17B, IL-17C, IL-17D, IL-17E, and IL-17F, were gradually discovered using sequence homology analysis. The first discovered IL-17 is termed IL-17A [18]. The sequences of IL-17A and IL-17F show approximately 55% homology, and they are often co-expressed. IL-17B, IL-17C, and IL-17D share 23–29% sequence homology, while IL-17E shares only 17% sequence identity with IL-17A, indicating that it is the most divergent subtype of the family [19, 20]. The IL-17 family members are secreted as disulfide-linked homodimers, with a molecular weight of 17–21 kDa.

The IL-17 cytokines exert their functions by activating their heterodimeric transmembrane receptors, which were first discovered in 1995 [21]. This discovery led to the successive identification of other homologous subunits, which are now termed as a new class of receptors: the IL-17R family, which consists of IL-17RA, IL-17RB, IL-17RC, IL-17RD, and IL-17RE, with IL-17RA as a common receptor. IL-17A and IL-17F bind to a dimeric IL17RA/RC complex, IL-17B and IL-17E bind to a dimeric 17RA/RB complex, and IL-17C binds to the IL-17RA/RE complex [22, 23]. However, the subunits of heterodimers specific to IL-17RD are yet to be identified [24–26].

**Fig. 1:**
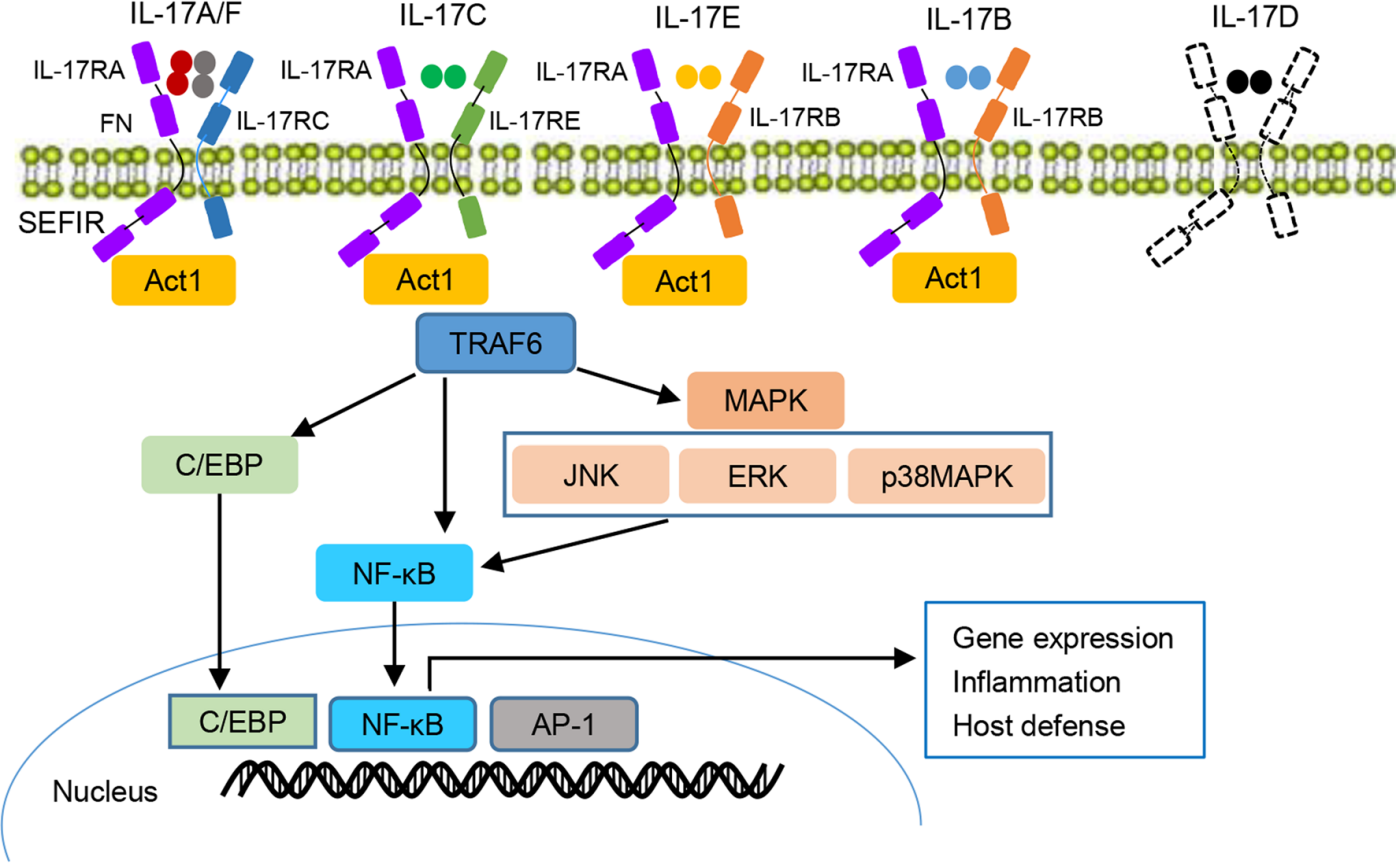
IL-17 cytokines, receptors, and signaling. The IL-17 family is composed of six members, IL-17A–F, while the IL-17 receptor family is composed of five members, IL-17RA to IL-17RE (IL-17RD not shown). IL-17 signaling is activated through the binding of the IL-17 receptor complex to the adaptor protein Act1, which has been determined in IL-17C to recruit TRAF6 to drive the activation of downstream signaling pathways, MAPK, C/EBP, and NF-κB, contributing to the target gene expression as well as mediating the host defense and inflammatory response. IL-17R interleukin 17 receptor, TRAF6 tumor necrosis factor receptor-associated factor 6, C/EBP CCAAT/enhancer-binding protein, MAPK mitogen-activated protein kinase, NF-κB nuclear factor-kappaB, Act1 NF-κB activator 1, JNK JUN N-terminal kinase. ERK extracellular signal-regulated kinase, FN fibronectin, AP1 activator protein-1

All receptor subunits are single transmembrane chains that contain an extracellular fibronectin (FN) III domain and share a cytoplasmic motif, similar expression to fibroblast growth factor (SEF)/IL-17R (SEFIR)Remove [27], which is conserved within the IL-17R family and is similar to the Toll/IL-1R homologous region (TIR domain) [28]. The SEFIR domain transduces the related signal to activate the adaptor protein nuclear factor-kappaB (NF-κB) activator 1 (Act1), followed by E3 ligase activation and the recruitment and ubiquitination of tumor necrosis factor receptor-associated factor 6 (TRAF6) [23]. TRAF6 subsequently activates multiple signaling pathways, such as members of the CCAAT/enhancer-binding protein (C/EBP) family and mitogen-activated protein kinase (MAPK) pathway, including JUN N-terminal kinase (JNK), extracellular signal-regulated kinase (ERK), and p38 MAPK, leading to the activation of NF-κB. In the nucleus, NF-κB induces the transcription of target genes directly or in combination with other transcription factors such as activator protein-1 (AP-1) (Fig. 1) [29–31].

## Pro-inflammatory function of IL-17A and IL-17F in pulmonary fibrosis

### IL-17A

IL-17A, the prototypic subtype of the IL-17 family, was first discovered in 1993 and named CTLA8 [32]. It was subsequently renamed IL-17 and, more recently, IL-17A.

IL-17A can be secreted by multiple cell types, including Th17, CD8^+^ T (Tc17), γδ T17, innate immune, and non-hematopoietic cells. It exerts its considerable influence via binding to the IL17RA/IL17RC heterodimer [22]. IL-17A production was reported to be significantly elevated in the bronchoalveolar lavage fluid of patients with IPF [33]. In IPF tissues, elevated IL-17 production can be observed in areas of active disease [34–36]. In these regions, IL-17 was originally reported to be mainly secreted by Th17 lymphocytes but was subsequently demonstrated to be produced by regenerating epithelium cells as well as immune cells, including γδ T cells, neutrophils, macrophages, and NK T cells [37]. IPF has been linked to repetitive epithelial cell injury, chronic inflammatory response, and activation of fibroblasts to secrete the extracellular matrix [38]. IL-17A is pivotal for multiple critical processes that promote fibrosis, including tissue repair, inflammatory response, and epithelial–mesenchymal transition (EMT) [39].

Multiple animal modeling studies have explored the role of IL-17A in driving early lung inflammation and fibrosis. During BLM injury, IL-17A expression is upregulated, contributing to the increased expression of other proinflammatory cytokines, including TNF-α, IL-1, IL-6, and TGF-β, as well as chemokines, such as IL-8, C-C motif chemokine (CCL) 2, C-X-C motif chemokine (CXCL) 1, and CXCL5, by endothelial cells and epithelial cells [40–42]. These molecules recruit certain inflammatory cells to the alveolar surface, and subsequent inflammation promotes pulmonary fibrosis [43]. Several studies have shown that BLM-stimulated IL-17 triggered significant neutrophilia and a marked increase in the levels of proinflammatory cytokines, such as IL-6 and IL-1β, and promoted pulmonary fibrosis [44]. Thus, IL-17A blockade by intraperitoneal anti-IL-17A alleviates the acute inflammatory and fibrotic features in mice [43]. Some studies have demonstrated that depletion of alveolar macrophages downregulates IL-17 responses to silica-induced early alveolitis and fibrosis by mediating the production of IL-23 and IL-1β [45].

IL-17A and its signaling cascade IL-17R subunits, as well as the post-translational modification of Act1, mediate tissue inflammation through various signaling pathways [44]. However, the receptor for IL-17A is also ubiquitously expressed in non-hematopoietic cells, including epithelial cells and fibroblasts, which play a vital role in the development of pulmonary fibrosis by EMT and differentiation into myofibroblasts, resulting in enhanced extracellular matrix deposition [46]. Additionally, IL-17 can play a role in lung fibrosis by suppressing autophagy in epithelial cells [47]. Consistent with this effect, IL-17A neutralization promoted autophagy, which presumably favored collagen resolution in the lungs [48]. In addition, in some studies, IL-17R production has been found to be elevated in fibroblasts following BLM challenge, and administration of exogenous IL-17 can accelerate fibroblast proliferation, leading to increased expression of α-smooth muscle actin (α-SMA) and collagen [49]. Mechanically, IL-17 stimulation of fibroblasts occurs via activation of the NF-κB/Act1 signaling pathway [50, 51].

### IL-17F

IL-17F shares the highest sequence homology (55%) and overlapping biological function with IL-17A [52], and the cell sources of IL-17F are similar to IL-17A, including Th17 cells, γδ T cells, CD8^+^ T cells (Tc17), and innate lymphoid cells. Additionally, IL-17F signaling occurs through the same IL-17RA/RC receptor complex. Thus, IL-17F expression is upregulated in multiple inflamed human tissues, and the importance of IL-17F in autoimmune and inflammatory diseases is becoming increasingly apparent [52, 53]. By inducing various cytokines, such as IL-6, and CXC chemokines in human tracheal epithelial cells, vein endothelial cells, inflammatory cells, and fibroblasts, IL-17F plays vital roles in allergic and chronic inflammatory lung disease [54]. Previous research has shown that IL-17F induces similar pathological phenotypes, such as recruitment of neutrophils and the same downstream inflammatory genes via the signaling components of IL-17RA, Act1, and TRAF6, which have been identified as key players in the IL-17A-mediated inflammatory response [55]. In addition, specific overexpression of IL-17F in the lungs of mice leads to infiltration of macrophages and lymphocytes as well as mucus production [52]. Currently, there is no direct evidence for the contribution of IL-17F to the progression of IPF; however, these observations related to IL-17A and inflammatory responses suggest that IL-17F may be an effective target for the treatment of IPF.

**Fig. 2:**
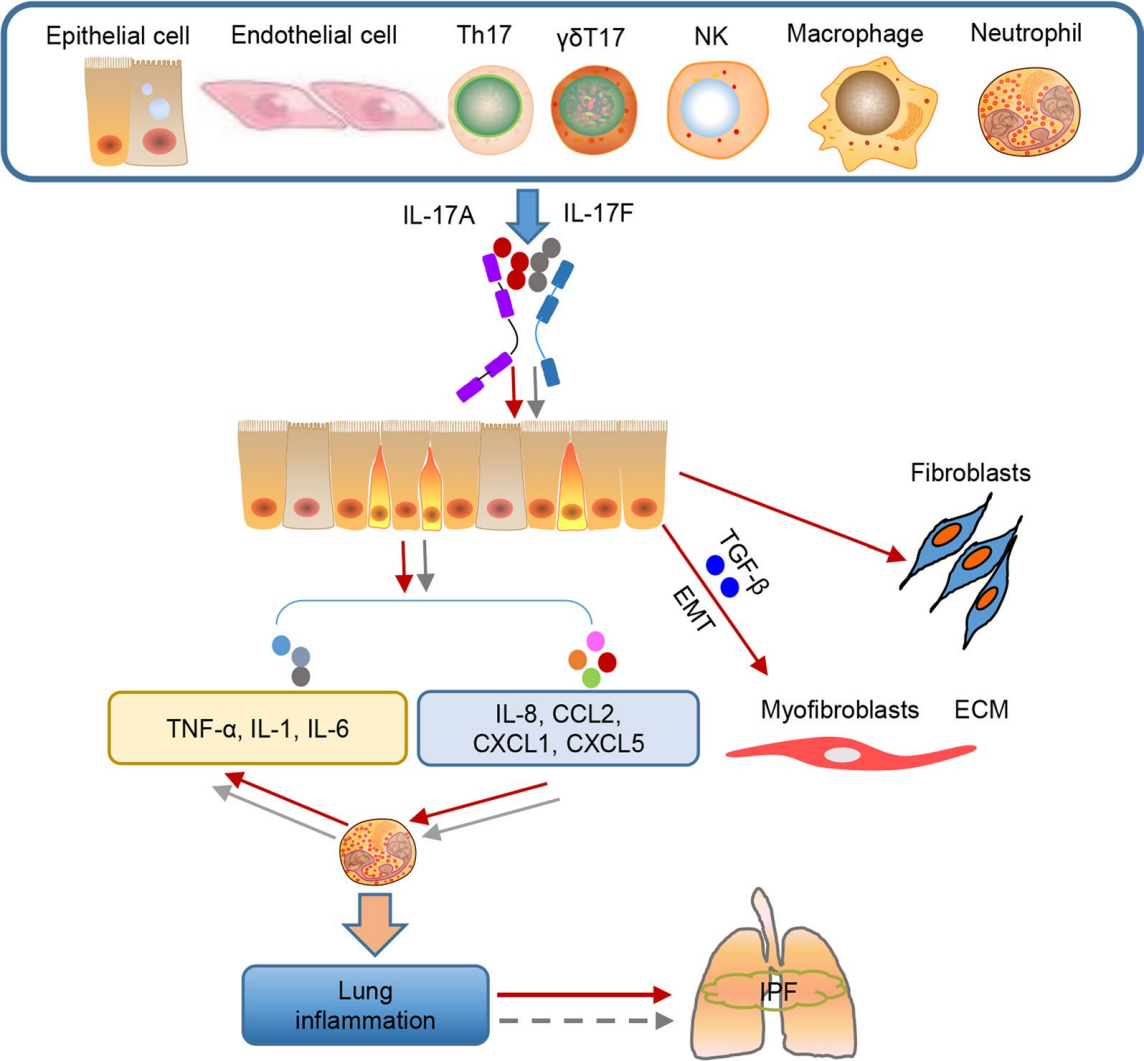
Main cellular sources and targets of IL-17A and IL-17F in IPF. IL-17A and F can be produced by Th17 cells and other immune cells such as γδ T cells, natural killer cells, macrophages, neutrophils, and non-hematopoietic cells such as epithelial and endothelial cells. IL-17A can contribute to pulmonary fibrosis by EMT, fibroblast proliferation, and differentiation to myofibroblasts. IL-17F stimulates lung inflammation by contributing to the infiltration of neutrophils, macrophages, and lymphocytes as well as promoting the expression of proinflammatory cytokines such as IL-6 and CXC chemokines; however, the role of IL-17F in IPF remains unclear. NK natural killer cells, Th17 T helper cell 17, TNF-α tumor necrosis factor-α, IL-1 interleukin-1, IL-6 interleukin-6, TGF-β transforming growth factor-β, IL-8 interleukin-8, CCL2 C-C motif chemokine 2, CXCL1 C-X-C motif chemokine ligand 1, CXCL5 C-X-C motif chemokine 5, EMT epithelial-mesenchymal transition, α-SMA α-smooth muscle actin, ECM extracellular matrix, IPF idiopathic pulmonary fibrosis

In summary, IL-17A derived from various cell types plays an important role in controlling the inflammatory response and fibrosis progression in IPF, and IL-17F may exert similar anti-inflammatory and anti-fibrotic effects, which need to be further explored (Fig. 2).

## Pro-inflammatory function of IL-17B and IL-17D in pulmonary fibrosis

### IL-17B

IL-17B was initially identified using a homology-based expressed sequence tag (EST) database [56, 57]. PCR and northern blot analysis showed that IL-17B expression was strong in the heart, testis tissues, and brain of mice, which have approximately 87.8% similarity to humans, and relatively low in other tissues, including in the liver, lungs, and skeletal muscle tissues [57]. IL-17B is functionally similar to IL-25 and elicits type 2 cytokine secretion from innate type 2 lymphocytes, NKT, and CD4^+^ CRTH2^+^ Th2 cells. Some reports revealed that this activity of IL-17B was dependent on the IL-17RA and IL-17RB receptor subunits [58]; however, it was shown to display a weak affinity of 7.6 nmol/L between IL-17B and IL-17RB [57].

Research on IL-17B has been limited; recent studies have shown that the epithelium weakly expresses IL-17B, whereas some connective tissue cells express abundant IL-17B, and neutrophils significantly express IL-17B [59]. Moreover, considerable amounts of IL-17B protein have been detected in naive and memory B cells, germinal center (GC) B cells, and neuronal cells.

**Fig. 3:**
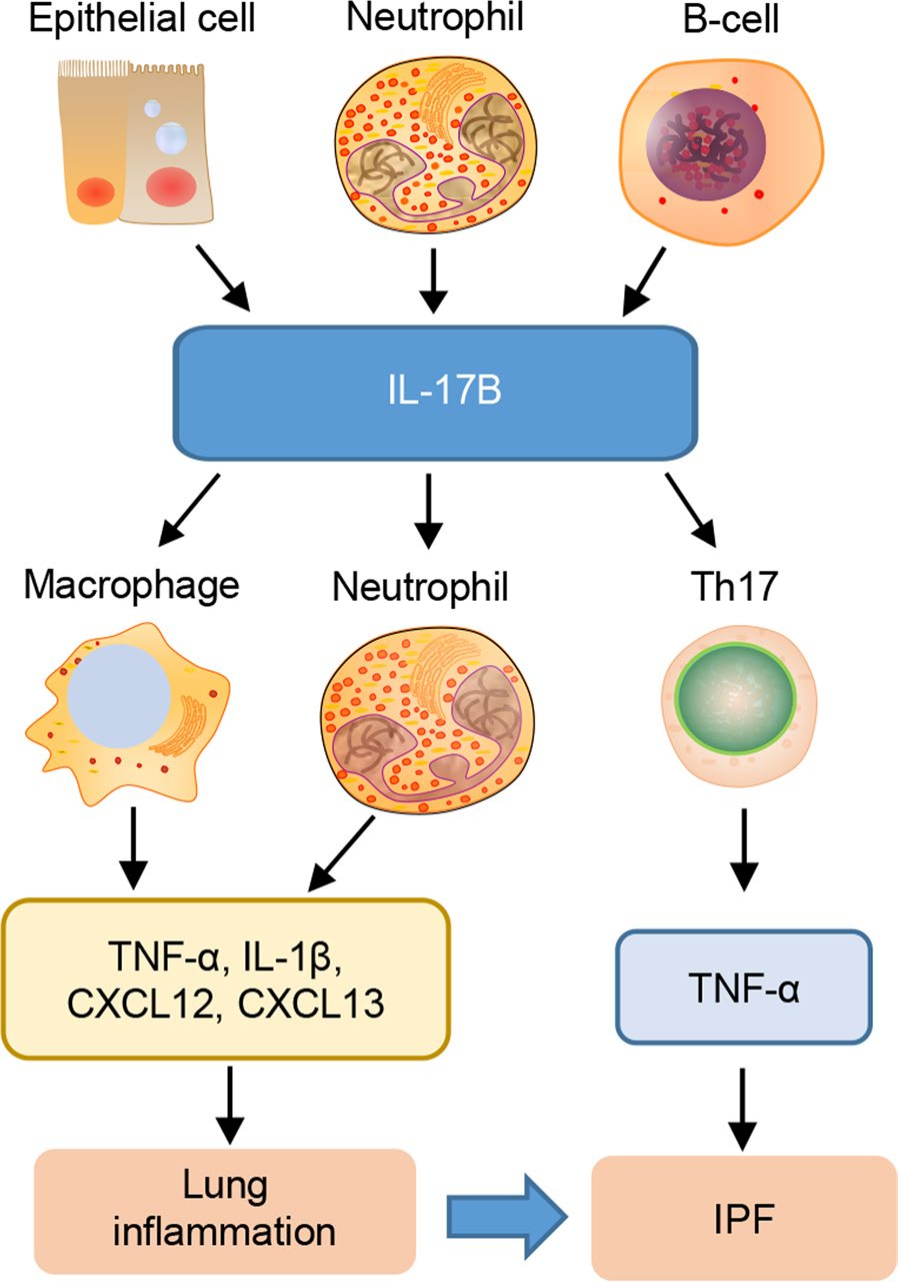
Main cellular sources and targets of IL-17B in IPF. IL-17B is mainly secreted from the epithelium, neutrophils, and B cells. It plays an important role in mediating lung inflammation by stimulating the expression of the inflammatory mediators IL-6, IL-23, TNF-α, IL-1β, and chemokines from macrophages, neutrophils, and Th17 cells. IL-17B induces lung fibrosis by cooperating with TNF-α to stimulate the secretion of Th17-cell-promoting genes and neutrophil-recruiting genes. Th17 T helper cell 17, TNF-α tumor necrosis factor-α, IL-1β interleukin-1β, CXCL12 C-X-C motif chemokine ligand 12, CXCL13 C-X-C motif chemokine ligand 13, IPF idiopathic pulmonary fibrosis

The function of IL-17B has not been thoroughly investigated, but several studies have revealed that its function partially overlaps with that of IL-17A and that it exhibits proinflammatory effects under certain conditions [60]. For example, IL-17B increases the production of the inflammatory mediators IL-6, IL-23, and IL-1α in the peritoneal exudate cells and 3T3 cell line [61] as well as the production of TNF-α and IL-1β in THP-1 cells, a cell line derived from human monocyte/macrophage cells [56]. IL-17B promotes the recruitment of C-X-C chemokine receptor (CXCR)4^+^ or CXCR5^+^ GC B cells to CXCL12 and CXCL13, and intraperitoneal administration of recombinant human IL-17B causes the migration of neutrophils to the peritoneal cavity and triggers the secretion of chemoattractive factors from multiple cell types [57]. Additionally, IL-17B can enhance IL-33-driven type 2 immune responses [62]. These proinflammatory functions suggest that IL-17B may influence the progression of IPF, which is prompted by early inflammation of the lung. However, no direct study identified this until Yang et al. [63] reported in 2019 that the expression of IL-17B was induced by dysregulated microbiota and that it induced lung fibrosis in a BLM-induced mouse model by interacting with TNF-α to stimulate the secretion of Th17-cell-promoting genes and neutrophil-recruiting genes (Fig. 3). Further studies are required to determine the detailed mechanism of action of IL-17B in cellular and IPF models.

### IL-17D

The IL-17D isoform of the IL-17 family, which maps to chromosome 13p11 in humans, discovered in 2002 displays 27% sequence identity with IL-17B [15]. The receptors and functions of IL-17D remain poorly understood. IL-17D mRNA is observed in a wide range of tissues, including the adipose tissue, lung, heart, pancreas, and brain. Intriguingly, IL-17D is detected only in B cells and resting CD4^+^ T cells among activated immune cells and rarely stimulates immune cells; it contributes to the secretion of proinflammatory factors through endothelial cells [64]. Knowledge of IL-17D in the regulation of pulmonary fibrosis remains limited, and further investigations are needed to understand the relationship between IL-17D-induced inflammatory response and pulmonary fibrosis development.

## Effect of IL-17C in pulmonary fibrosis from its various inflammatory function

IL-17C was first discovered by Li et al. [56] and exhibited low sequence homology (23%) with IL-17A. IL-17C has been shown to be expressed in various cells, including epithelial cells, CD4^+^ T cells, DCs, and macrophages. IL-17C produced by epithelial cells has been reported to be associated with antimicrobial activity [65]. Stimulation of respiratory epithelial cells with whole bacteria in vitro and bacterial challenge in a mouse model rapidly induced IL-17C expression [66], leading to an enhanced lung inflammatory response [67].

Several studies have shown that IL-17C acts through an IL-17 receptor complex, consisting of the common IL-17RA subunit, which is shared with IL-17A, IL-17F, and IL-17B, and the specific IL-17RE subunit [23, 68]. IL-17RE has been shown to be located mainly on epithelial cells and Th17 cells. Th17 cells can produce elevated amounts of IL-17A, IL-17F, and IL-22 stimulated by IL-17C, indicating that IL-17C might promote cell differentiation or maintenance [68]. Additionally, IL-17RA and IL-17RE have been reported to be expressed on other types of cells; they enable IL-17C to play an important role in host defense, autoimmune, and inflammatory pathologies including lung inflammation [62, 63]. For example, adoptive transfer of IL-17C-transduced CD4^+^ T cells leads to significantly exacerbated collagen-induced arthritis [68]; IL-17C induces the release of TNF-α and IL-1β from THP-1, a monocytic cell line [61]; and adenoviral challenge of IL-17C in the lung triggers neutrophil recruitment [69].

A specific NF-κB binding site has been identified in the promoter region of IL-17C, and IL-17C production is dependent on NF-κB activation. Intriguingly, IL-17C binds to its receptor subunits IL-17RA and IL-17RE, activating the Act1 complex, which in turn activates the NF-kB and MAPK signaling molecules, contributing to the secretion of inflammatory factors and lung destruction [70].

**Fig. 4:**
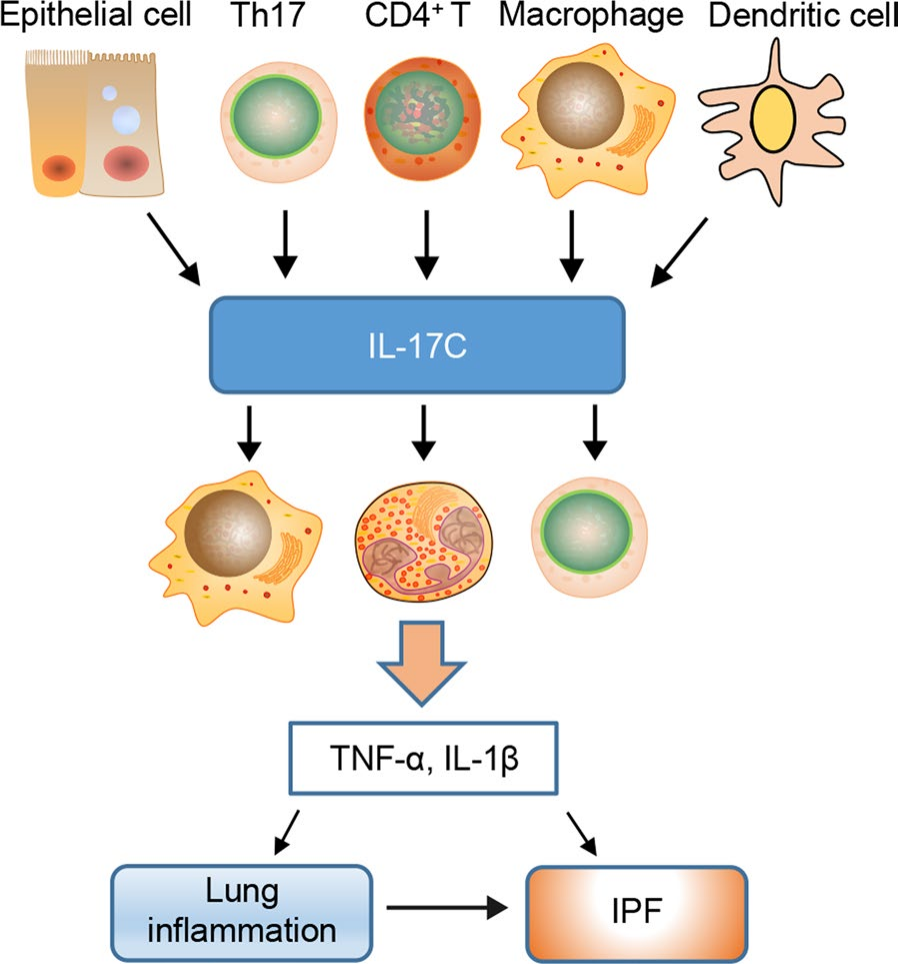
Main cellular sources and targets of IL-17C in lung inflammatory response. IL-17C is expressed in various cells, including epithelial cells, Th17 cells, CD4^+^ T cells, DCs, and macrophages. It acts on multiple types of cells, including CD4^+^ T cells, macrophages, and neutrophils, playing an important role in lung inflammation. Definitive evidence has not been reported for the role of IL-17C in regulating pulmonary fibrosis. Th17 T helper cell 17, TNF-α tumor necrosis factor-α, IL-1β interleukin-1β, IPF idiopathic pulmonary fibrosis

The function of the IL-17C isoform in the progression of IPF has not been extensively investigated, and pulmonary fibrosis has been widely demonstrated to be driven by epithelial cell injury and the inflammatory response during wound healing, which is associated with the function of multiple immunocytes [71, 72]. Early lipopolysaccharide-induced lung injury can be established as a model of pulmonary fibrosis. Reports of related injury of epithelial cells, release of proinflammatory mediators, and IL-17C-induced neutrophil recruitment identified its important roles in lung inflammation. Boosted IL-17 production of IL-17C in IL-33-, NTHi- and cigarette smoke-induced lung inflammation has been recently reported [73, 74]. All of these findings suggest that IL-17C might be a key cytokine in the pathogenesis of pulmonary fibrosis (Fig. 4). Nevertheless, definitive evidence is yet to be provided, and the underlying mechanisms of action of IL-17C in regulating pulmonary fibrosis remain to be elucidated.

## Protective and promotive roles of IL-17E in inflammatory response and fibrosis in lungs

**Fig. 5:**
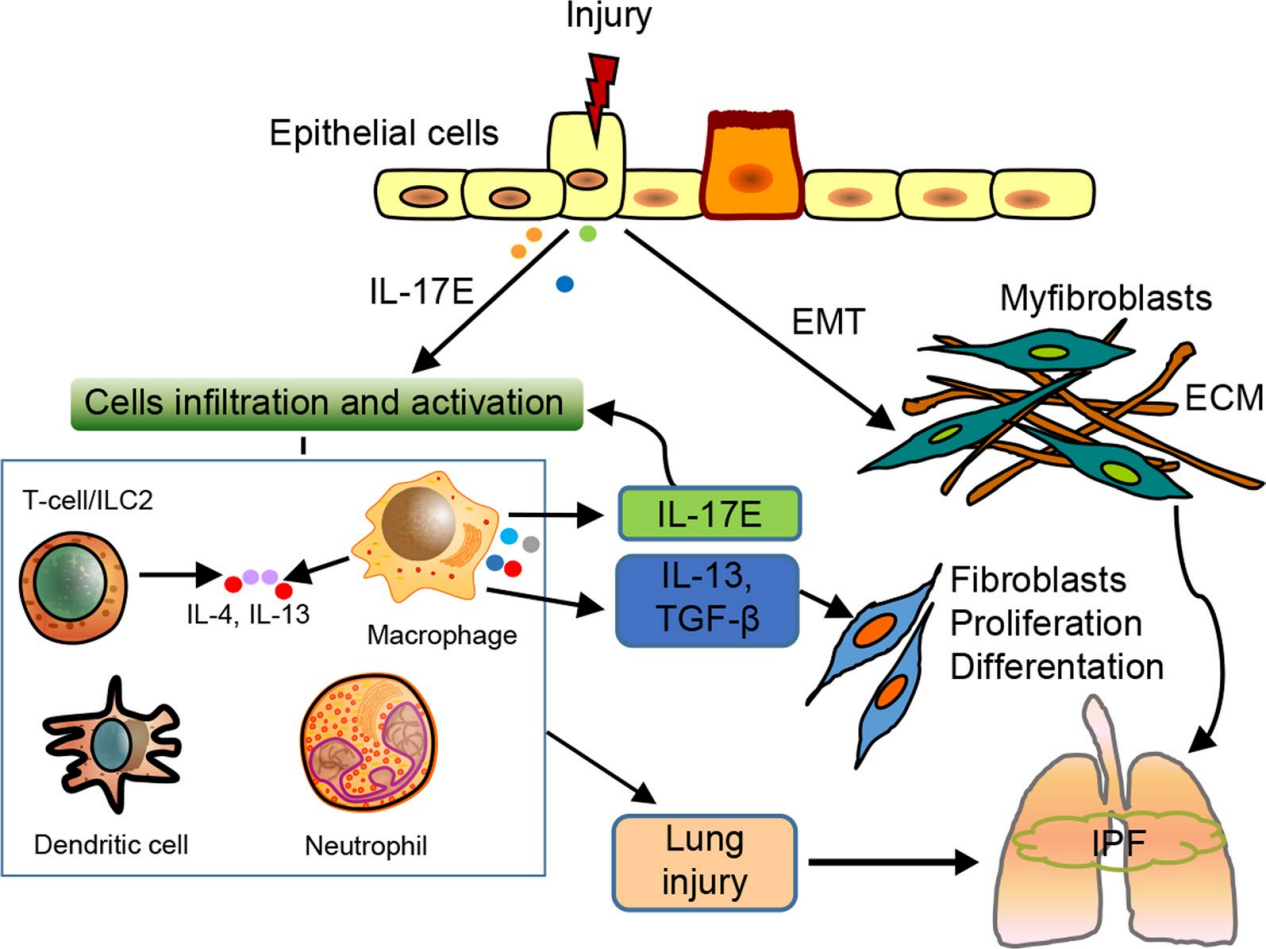
Involvement of IL-17E in IPF. IL-17E is produced from injured epithelial cells. It acts on T cells, ILC2s, alveolar macrophages, DC and neutrophils, which can stimulate the secretion of cytokines such as IL-17E, IL-13, and TGF-β, drive lung fibrosis by mediating EMT, as well as recruit and activate lung fibroblasts. IL-17E can additionally activate a series of cell types, leading to lung inflammation, which is critical in the development of IPF. ILC2s type 2 innate lymphoid cells, IL-4 interleukin-4, IL-13 interleukin-13, TGF-β transforming growth factor-β, EMT epithelial–mesenchymal transition, ECM extracellular matrix, IPF idiopathic pulmonary fibrosis

IL-17E, commonly known as IL-25, was originally discovered in Th2 cells in 2001 and later classified as an isoform of the IL-17 family. Later, scientists observed that IL-17E is secreted not only by Th2 cells but also by epithelial cells, endothelial cells, T cells, alveolar macrophages, ILC2s, DCs, eosinophils, and basophils, all of which are associated with inflammatory responses [75, 76]. IL-17E signals through a heterodimeric receptor composed of IL-17RA and IL-17RB, both of which are essential for cytokine expression in target cells during inflammatory disorders. IL-17E initiates allergic airway diseases by producing excessive cytokines, such as IL-4, IL-5, and IL-13 [77]. Remarkably, IL-17E plays protective role in some inflammatory responses, such as parasitic infection and dextran sulfate sodium-induced colitis [78, 79]. A few recent studies have revealed that IL-17 is involved in the development of lung fibrosis. Xu et al. [80] reported augmented levels of IL-17E (IL-25) and its receptor IL-17RB in the lung tissues of patients with IPF and showed that they drove lung fibrosis by mediating the EMT of alveolar epithelial cells as well as recruiting and activating lung fibroblasts. Hams et al. [81] observed a population of type 2 innate lymphoid cells (ILC2s) and increased production of IL-25 in the lungs of patients with IPF and reported that IL-25 promoted the release of IL-13 from ILC2s, which triggered collagen deposition during the IPF process. The relationship between IL-17E and IPF is summarized in Fig. 5. Further investigation is required for identifying other mechanisms by which IL-25 regulates IPF.

## Conclusions

The potential role of IL-17 isoforms in pulmonary fibrosis has attracted increasing interest over the last few years. Several members of the IL-17 family have been identified to be promptly secreted by either immune cells or non-hematopoietic cells, playing vital roles via cascade signaling in key stages of IPF progression from the early inflammatory response to the late fibrotic process. Other undefined isoforms have been implicated in mediating protection against antimicrobial infections and inflammatory responses (Table 1). The current challenge is to precisely define the interplay among these IL-17 isoforms and determine their mechanisms of action in the pathogenesis of IPF.

**Table 1:** Preclinical and clinical data of IL-17 family members in pulmonary inflammation and fibrosis

IL-17 family member	Expression changes in inflammatory lung	Preclinical role in PF	Clinical role in PF
IL-17A	Elevated in BLM, IL-33 and LPS-induced lung inflammation [36, 44]	Contributing to fibrosis by promoting proinflammatory cytokines [39, 43]; triggering neutrophilia [44]; promoting EMT [46], accelerating fibroblasts proliferation, differentiation [46, 49]	Elevated in: lung of RA-ILD patients [35];airways of cystic fibrosis patients [34]
IL-17B	Expression of IL-17B was induced by dysregulated microbiota [63]	Elevated in BLM-induced PF mouse model by regulating Th17-cell-promoting genes and neutrophil-recruiting genes [63]	No data
IL-17C	IL-17C contributes to NTHi-induced inflammation and lung damage [74]	Remains limited	No data
IL-17D	Remains limited	Remains limited	No data
IL-17E	Protective roles in inflammatory response [75, 76]	Drove lung fibrosis by mediating EMT; recruiting and activating lung fibroblasts [80]; promotes IL-13 from ILC2s; triggering collagen deposition [81]	Elevated in lung of IPF patients [80]
IL-17F	Recruitment of neutrophils, macrophages, lymphocytes; promotes inflammatory cytokines [52, 55]	No direct evidence for the progression of IPF	

*IL-17* interleukin 17, *IPF* idiopathic pulmonary fibrosis, *BLM* bleomycin, *IL-33* interleukin-33, *LPS* Lipopolysaccharides, *NTHi* nontypeable *Haemophilus influenzae*, *PF* pulmonary fibrosis, *EMT* epithelial-mesenchymal transitions, *Th17 cells* T helper cell 17, *IL-13* interleukin-13, *ILC2s* group II innate lymphoid cells, *RA* rheumatoid arthritis, *ILD* interstitial lung disease

Drugs that target IL-17 signaling are presently available in the market; brodalumab inhibits the human IL-17A receptor, and secukinumab and ixekizumab block IL-17A itself [82, 83]. These three FDA-approved IL-17 inhibitors have demonstrated marked efficacy in patients with inflammatory diseases, such as ankylosing spondylitis and psoriatic arthritis [83–85]. Through additional preclinical data and clinical trials that explore the efficacy of IL-17 neutralization for IPF, the usefulness of IL-17-related monoclonal antibodies in IPF should be considered. Furthermore, extensive information on the precise signaling mechanisms and biological functions of each IL-17 member would pave the way for therapeutic interventions that selectively target specific IL-17 members for the treatment of IPF in humans.

## Data Availability

Not applicable.

## Abbreviations

AP-1: Activator protein-1
BLM: Bleomycin
C/EBP: CCAAT/enhancer-binding protein
CXCR: C-X-C chemokine receptor
EMT: Epithelial–mesenchymal transition
ERK: Extracellular signal-regulated kinase
EST: Expressed sequence tag
FN: Fibronectin
GC: Germinal center
GM-CSF: Granulocyte macrophage-colony stimulating factor
IL: Interleukin
IL-17R: IL-17 receptor
ILC2: Type 2 innate lymphoid cells
IPF: Idiopathic pulmonary fibrosis
JNK: JUN N-terminal kinase
LPS: Lipopolysaccharide
MAPK: Mitogen-activated protein kinase
NF-κB: Nuclear factor κB
SEF: Similar expression to fibroblast growth factor
SEFIR: SEF/IL-17R
α-SMA: α-smooth muscle actin
TNF-α: Tumor necrosis factor-α
TRAF-6: Tumor necrosis factor receptor-associated factor 6
